# ROBO4 deletion ameliorates PAF-mediated skin inflammation via regulating the mRNA translation efficiency of LPCAT1/LPCAT2 and the expression of PAF receptor

**DOI:** 10.7150/ijbs.35797

**Published:** 2020-02-04

**Authors:** Xiaoqiang Xiao, Xi Zhuang, Ciyan Xu, Haoyu Chen, Weiquan Zhu, ChiPui Pang, Mingzhi Zhang

**Affiliations:** 1 Joint Shantou International Eye Center, Shantou University and the Chinese University of Hong Kong, Shantou, China.; 2 Department of Ophthalmology & Visual Sciences, the Chinese University of Hong Kong, Hong Kong, China; 3 Department of Medicine, Program in Molecular Medicine, University of Utah

**Keywords:** ROBO4, PAF, hair loss, oxidative phosphorylation, ribosome

## Abstract

The diminished level of platelet-activating factor acetylhydrolase (PAFAH) in milk causes an enhanced level of platelet activating factor (PAF) in the skin, leading to a severe hair loss phenotype during neonatal pup's lactation. The deletion of very-low-density-lipoprotein receptor (VLDLR) prevents the expression and secretion of PAFAH. Here we revealed that deletion of Roundabout 4 (ROBO4) in mice ameliorated hair loss phenotype via reducing PAF concentration in skin. As a consequence, the neonatal pups with ROBO4 deletion lactated by mother with VLDLR deletion showed normal hair phenotype during lactation. In details,ROBO4 deletion reduced the protein but not mRNA expression of two PAF synthetic enzymes LPCAT1/LPCAT2 in macrophage as well as the expression of PAF receptor in both macrophage and ocular tissue, but increased PAFAH protein in serum. On the other hand, RNA expression profile analysis in macrophages revealed that the genes involving in oxidative phosphorylation and ribosome obviously decreased their expression in response to ROBO4 deletion. Moreover, through High Performance Liquid Chromatography (HPLC) analysis, we found that ATP concentration also reduced in ROBO4 deletion macrophages. Because ribosome and energy are very important factors for the mRNA translation, we then tested whether ROBO4 deletion affects LPCAT1/LPCAT2 mRNA translation using polyribosome assay. As expected, the mRNA level of LPCAT1/LPCAT2 significantly decreased in polyribosome in ROBO4 deletion macrophage comparing to that of wild type. Additionally, mice with ROBO4 deletion suppressed LPS-induced IL-6 expression as well as the phosphorylation of p44/42 and p65, but enhanced the AKT phosphorylation. Collectively, ROBO4 deletion alleviates PAF- and LPS-mediated inflammation. And above results also indicate PAF signal might be a crosstalk point of ROBO4- and VLDLR-activated pathways.

## Introduction

Platelet-activating factor (PAF, 1-O-alkyl-2-acetyl-sn-glycero-3-phosphocholine), a lipid mediator, has been implicated in the inflammation, asthma, anaphylaxis 1, 2. It was also reported that PAF induced transient blood-brain barrier opening and facilitated the penetration of edaravone into the brain 3. Recently, PAF was confirmed to be a regulator of retinal neovascularization 4. PAF can be released by many types of cell such as platelets, monocytes/ macrophages, neutrophils and endothelial cells 3, 5. Mother's milk contains immune-defensive factors such as platelet-activating factor acetylhydrolase (PAFAH) 6 and thus can protect newborn baby through nursing. PAFAH, a target of VLDLR signal, hydrolyzes PAF and protects the PAF-mediated inflammation in the skin of pups 6. PAF binding to PAF receptor activates many physiological events including inflammation, angiogenesis and tumorgenesis 4, 7, 8. PAF signal balance can be elaborately regulated by controlling PAF production, PAF hydrolyzation and PAF/PAF receptor pathways 7. On the other hand, ROBO4 can stabilize vasculature and deleting ROBO4 promote retinal neovascularization 9, 10. It was also reported that ROBO4 regulates the localization of hematopoietic stem cell to bone marrow niches via cooperating with CXCR4 11. Recently, ROBO4 also involved in LPS induced endothelial inflammation 12. Those observations indicate that ROBO4 participates in various biological functions.

In this study, we revealed that ROBO4 regulates PAF-mediated inflammation via interfering with its synthesis, signal pathway activation and degradation pathway. Moreover, LPS-induced inflammation was also blocked by ROBO4 deletion.

## Materials and Methods

### Reagents and antibodies

All the reagents were purchased from Sangon Biotech (Shanghai, China) and Sigma (Shanghai, China). Adenosine triphosphate (ATP) (purity > 95%) was purchasedfrom Dalian Meilun Biotechnology, China. Anti-LPCAT1 and Anti-LPCAT2 antibodies were purchased from NOVUS, USA. Anti-PAF receptor antibody was purchased from Abcam, USA. Antibodies for protein phosphorylation were all purchased from Cell Signaling Technology,USA.

### Animals

Vldlr-/- mice were purchased from Jackson Laboratory (Stock No: 002529) (Bar Harbor, ME) and backcrossed to C57BL/6 for at least 3 generations. ROBO4-/- mice 10 were kindly gift from Prof.Dean Y Li Laboratory (USA). VLDLR deletion (VLDLR-/-) mice crossed with ROBO4 deletion (ROBO4-/-) mice, and produced the F1 generation mice with VLDLR-/+VS ROBO4-/+ genotype. Then, the F1 generation mice self-crossed and produced the F2 generation mice. PCR was used to identify the genotypes of mice. Mice were fed with standard rodent chow. For cross-fostering experiments, different genotypes of mice pups were separated and nursed with the designated genotype's mother during the whole lactation period. For LPS I.P-injected experiments, C57BL/6 mouse (4 mice) or ROBO4 -/- mouse (4 mice) (ages: two months) were used and tissues were taken out after 24 hours injection. All of experimental procedures were approved by Experimental Animals Administration Committee of Joint Shantou International Eye Center Shantou University & the Chinese University of Hong Kong.

### Macrophage differentiation and total RNA extraction

For macrophage differentiation, bone marrow cells or splenocytes were isolated from Vldlr-/- , ROBO4-/- mice or WT mice, and cultured in DMEM containing 10% FBS and 20ng/ml M-CSF (Sigma, USA) for 12 days with a change of medium every 4 days. On day 12, cells were harvested or treated with U0126 (1μM) for another 24 hours. Part of the cells were lysated with 1×NP40 buffer (containing complete protease inhibitor) for Western-blotting analysis with designated antibodies. The remaining cells were used for the total RNA extraction with TRIzol reagent (Invitrogen, USA). The cDNAs were produced by reverse-transcription kit (Invitrogen, USA) with total RNA (2μg) as a template. Realtime-PCRs were then performed using the SYBR green Realtime PCR kit (Takara, Dalian China) with designated primers set listed in table 1 in an Applied Biosystems 7500 Real-Time PCR machine. The total RNA of skin was used the same protocol as in the macrophage and qPCR system was also the same.

### RNA sequencing and bioinformatics analysis

Spleen and bone marrow macrophage cells differentiated via 20ng/ml M-CSF (Sigma,USA) from Robo4 knockout or wild type C57 mice were lysated with Trizol and sequenced by commercial available service(BGI,Huada,China). Poly (A) containing RNAs were enriched by magnetic beads carrying oligo(dT). Sequencing libraries were generated after first strand cDNA synthesis and adaptor ligation. Message RNA(mRNA) sequencing was carried out on an Illumina HiSeq X ten platform that produced 2×100 bp paired-end (PE) raw reads (Novogene Bioinformatics Technology Co.Ltd). The raw reads were treated to produce clean reads. The clean sequence tags were mapped to the UCSC mm10 reference genome. PE clean reads were aligned to the reference genome using TopHat v2.0.12. Gene expression levels were measured by transcript abundance. The parameter FPKM (Fragments Per Kilo base of exon per Million fragments mapped) was used to compare the gene expression in different genes and groups. Statistically significant differentially expressed genes from RNA-seq data were obtained by applying a cut-off threshold of FDR ≤ 0.05 (5%). The Geneontology(GO) and biochemical pathways used in the analysis in this paper were obtained from PANTHER, DAVID and BINGO software. Using the web-based annotation tool DAVID v 6.7 (http://david.abcc.ncifcrf.gov/) to cluster differentially regulated genes by their common functionality. Clustering enrichment thresholds required DAVIDEASE scores such that the P-value was < 0.05. To graphically display associations between two epileptic genes, network analysis was done to show both direct and indirect interactions using STRING and GeneMania software.

### PAF analysis by Mass Spectrometry

The concentration of PAF in skin was detected by LC-MS/MS analysis. The detailed protocol is described in the previous paper 6.Briefly, the total lipids of skin tissues were extracted with chloroform and methanol. d4-PAF-C16 and d4-PAF-C18 standards (Cayman Chemical,USA) were used for the internal control of extraction efficiency. A reverse phase separation of the lipids was performed on a Gemini C18 column (Phenomenex) with mobile phase A(0.1% formic acid v/v) and mobile phase B(100% acetonitrile, 0.1% formic acid v/v).Then elution and re-equilibration steps to the column were performed with mobile phase B.MS/MS quantification of PAF-C16, d4-PAF-C16, PAF-C18, d4- PAF-C18 was performed on an Agilent 6410 triple quadrupole mass spectrometer in multiple reaction monitoring (MRM) mode using electrospray ionization in positive ion mode. The level of each endogenous PAF was normalized by the internal control.

### Enzyme-linked immunosorbent assay (ELISA) and Western blotting

The serums from ROBO4 deletion and wild type mice were collected for cytokine analysis by ELISA. The PAFAH ELISA kits were obtained from biocompare Biological Technology Co. Ltd. (USA). The detection steps were strictly based on the manufacturer's instructions provided within the kit. Western blotting was performed in a standard protocol with the designated antibodies. Total proteins were extracted with 1×RIPA or NP-40 buffer containing the cOmplete™, EDTA-free Protease Inhibitor Cocktail (Roche,USA) and quantified with the BCA kit. GAPDH was used as a loading control.

### Polyribosome assay

Mouse macrophage cells were grown to 80% confluency. Cycloheximide(100 μg/ml) was used to treated the cells for 10 min at 37°C before lysis. Then cells were lysed in 300 μl of lysis buffer (10 mM HEPES pH 7.4, 150 mM KCl, 10 mM MgCl2, 1% NP-40, 0.5 mM DTT, 100 μg/ml cycloheximide) following the wash with ice-cold PBS (supplemented with 100 μg/ml cycloheximide) for two times. The lysates were centrifuged (12,000 *g*, 15 min, 4°C) to remove the nuclei and the membrane debris. The supernatant was then slightly added into the prepared sucrose gradient(10-50%[w/v]), supplemented with 10 mM HEPES pH 7.4, 150 mM KCl, 10 mM MgCl2, 0.5 mM DTT, 100 μg/ml cycloheximide) and centrifuged (160,000 *g*, 120 min, 4°C) in an SW41Ti rotor (Beckman).RNA from polysome fractions were extracted with Trizol(Invitrogen,USA) and the first strand of cDNA was synthesized with reverse transcriptional kit(Takara,China). After cDNA synthesis, qPCR was used to assess the mRNA amount of designated genes, GAPDH as an internal control.

### Tissue ATP assay

Differentiated macrophages isolated from Robo4 deletion or wild type mice (p21) were used for the ATP extraction. Cells were lysated with 1×NP40 lysis buffer (50 mM Tris, pH 7.4, 250 mM NaCl ,5 mM EDTA ,50 mM NaF, 1 mM Na3VO4, 1% Nonidet™ P40 (NP40), 0.02% NaN3) supplemented with 1mM PMSF (Sigma) and complete protease inhibitor cocktail (Roche). After centrifuged (12000g, 15 min, 4°C), the supernatants were injected into Agilent 1260 Infinity HPLC system 1260. The ATP was separated by Polaris 5 C18-A column (Agilent, 2.1 mm × 150mm, 5 *μ*m) with temperature set at 25°C, detection wavelength at 258 nm, and isocratic elution composed of mobile phase A (methanol, 0.6%) and mobile phase B (99.4%) at a flow rate of 1 mL/min. The concentration of ATP was showed as peak area. The standard ATP was used for the position reference of sample ATP.

## Results

### ROBO4 deletion alleviates the transient alopecia of neonatal pups

**Figure 1:**
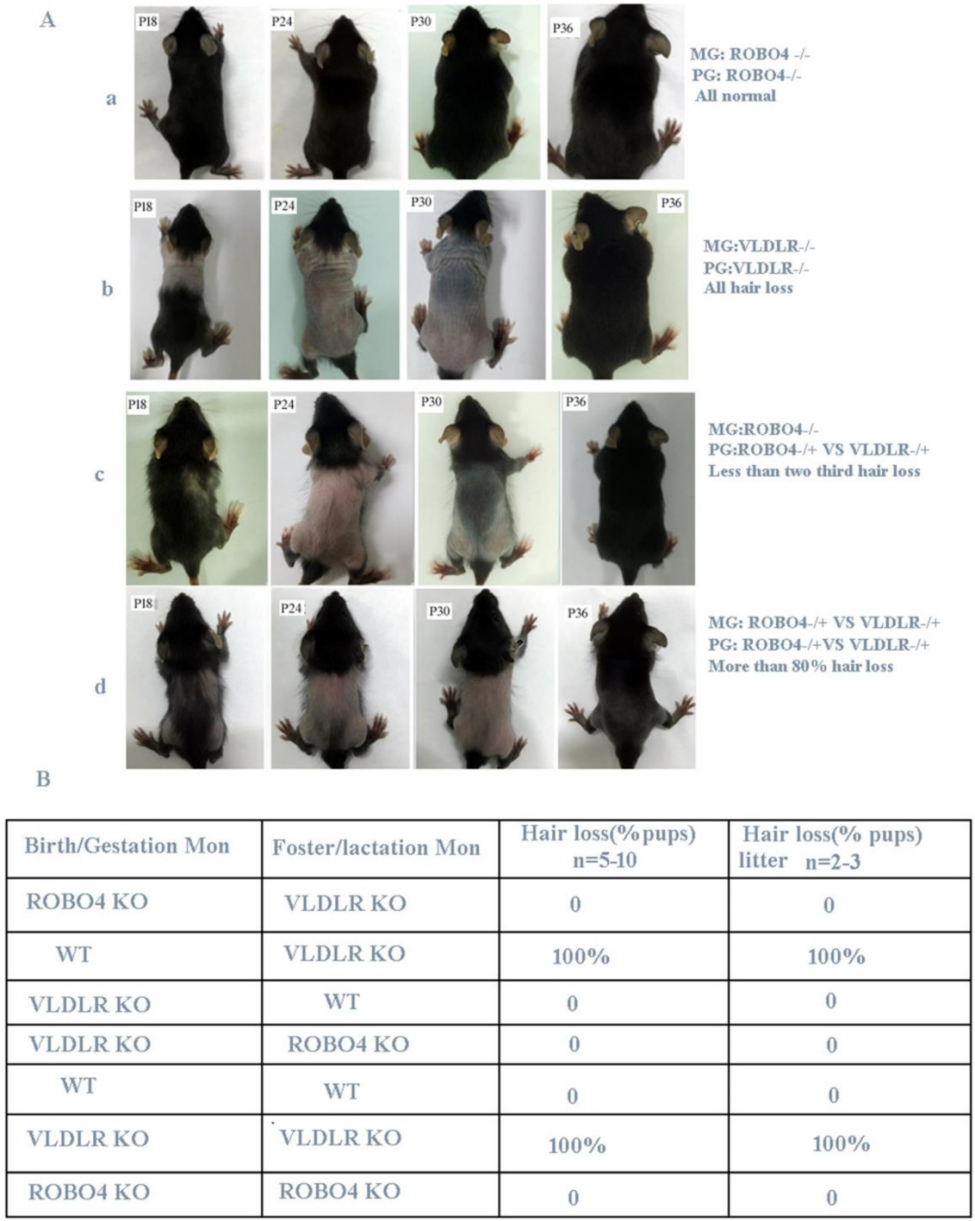
** ROBO4 deletion alleviates the transient hair loss phenotype of neonatal pups lactated by VLDLR deleting mothers. A,** Hair loss phenotype in different genotypes of neonates after crossing. The number of neonates in each group is more than 24. **B,** Hair loss phenotype of cross-feeding among different genotypes of mice; VLDLR-/-, ROBO4-/-: double isoforms deletion; VLDLR-/+, ROBO4-/+: single isoform deletion; Birth/Gestation Mon: neonates produced from designated genotype mother; Foster/Lactation mon: lactating mother's genotype.

Previous research revealed that VLDLR deletion leads to the reduction of PAFAH secretion in milk, thus causes PAF-mediated hair loss for neonatal pups if lactated by the same genotype's mothers 6. However, if nursing the normal neonatal pups with VLDLR deletion mother, they also showed a completely hair loss phenotype. The author thus concluded the hair loss phenotype occurred in neonatal pups is only determined by the mother's genotype 6. Mice with ROBO4 deletion causes vascular permeability and neovascularization 10 and their neonates show a normal skin during lactation. So we used the hair loss phenotype to observe the potential crosstalk between ROBO4 and VLDLR signal pathways. Firstly, we crossed ROBO4 deletion mice with VLDLR deletion mice to produce first generation (F1) mice. Then, the mice from F1 generation self-crossed and produced the second generation (F2) hybridization mice. We observed that a less than two-thirds of lactating F1 generation neonates showed a transient hair loss phenotype when nursed by mothers with ROBO4 deletion (Figure 1A, the third panel).Also, we observed that more than 80% of F2 generation neonates showed a transient hair loss when they were lactated by mother from F1 generation (Figure 1A, the last panel). However, most of the neonates with ROBO4 and VLDLR double knockout were also observed a severe hair loss phenotype during lactation (data not showed). Those results showed ROBO4 deletion disturbed the VLDLR-mediated hair loss phenotype of neonates, whereas ROBO4 deletion could not completely reverse this phenotype. To further characterize this phenomenon, we used the mice with different genotypes, including wildtype (WT), VLDLR KO and ROBO4 KO, via cross-fostering. We surprisingly found all neonates with ROBO4 deletion showed completely normal hair phenotype when nursed by mothers with VLDLR deletion (Figure 1B). On the other hand, the neonates with VLDLR deletion also showed normal hair nursed by mother with ROBO4 deletion, which was similar with the results of wild type (Figure 1B). As previous report, the protein of PAFAH significantly decreased in the milk of VLDLR deletion lactating mother, leading to PAF-mediated hair loss phenotype for their neonates. Therefore, those results indicate ROBO4 deletion might somehow affect PAF-balance in skin.

### ROBO4 deletion reduced PAF production in skin and the expression of LPCAT1/LPCAT2 as well as PAF receptor in macrophage

**Figure 2:**
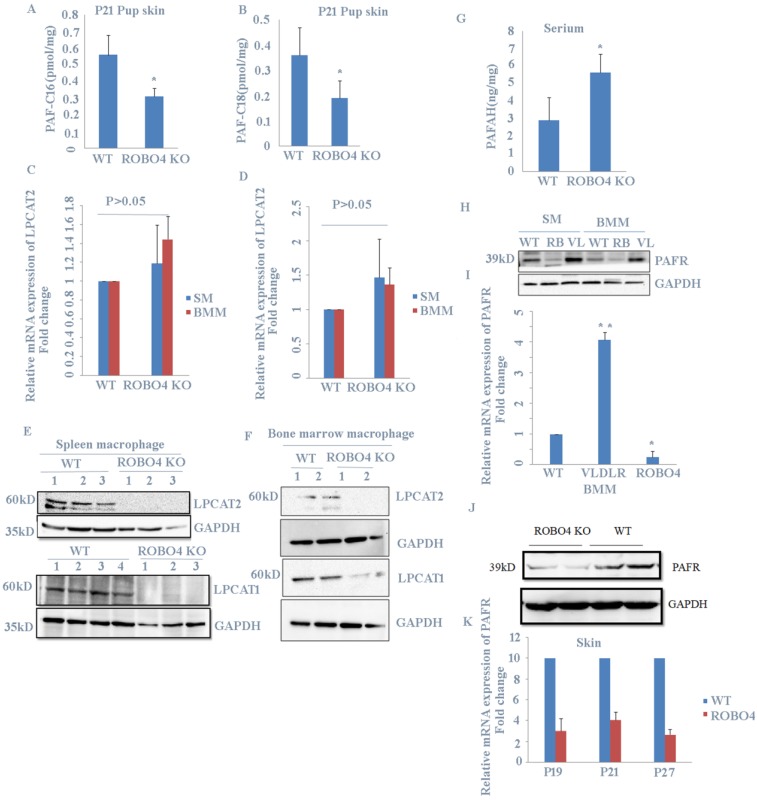
** ROBO4 deletion suppressed PAF production and expression of PAF receptor. A,B,** PAF levels were increased in the skin of the pups nursed by ROBO4 KO mothers compared to that nursed by WT mothers(Control). Tissue lipids were analyzed by MRM LC-MS/MS to quantify PAF-C16 (left)(A) and PAF-C18 (right)(B) levels (n=3). The results were normalized to d4-PAF-C16 or d4-PAF-C18 internal control, respectively. P,postnatal day. **C,D,** The mRNAs expression of PAF synthases,LPCAT1(C) and LPCAT2(D) were unaltered in the ROBO4 KO BM macrophage(BMM,n=4), and Spleen macrophage(SM,n=4) compared to WT macrophage(n=4); 1×10^6^ macrophages were differentiated from 1.5×10^6^ bone marrow cells or 5×10^6^ splenocytes of ROBO4 KO mice or WT control mice (2 month old).**E,F,** the proteins expression of LPCAT1(E,F low panel) and LPCAT2(E,F up panel) in both spleen(E) and bone marrow (F)macrophage were observably decreased in ROBO4 KO macrophage. GAPDH was used as a loading control. The macrophage differentiated manner was the same as in C.D. **G,** Serum level of PAFAH was elevated in the pups nursed by ROBO4 KO mothers compared to the pups nursed by WT control mothers (n=8, P21).**H,I,** The protein (H) and mRNA(I) expression of PAFR diminished in ROBO KO but elevated in VLDLR KO macrophage. WT, wild type; RB,ROBO4 deletion; VL,VLDLR deletion. The macrophage differentiated manner was the same as in C.D. GAPDH was used as a loading control. **J,K,** The protein and mRNA expression were also down regulated in ROBO4 deletion skin tissues compared to WT(wild type). Skin tissues from ROBO4 deletion or WT mice( six mice /each genotype, randomly classified into two groups) were mixed for the total protein extraction and detected by PAFR specific antibody(J);the mRNA expression of PAFR in skin tissues was tested in ROBO4 deletion or WT mice at postnatal 19(P19),postnatal 21(P21) and postnatal 27(P27) (K). Statistical analyses were performed with Student's t-Test and are shown as mean ± standard deviation;*, p<0.05; Experiment repeated at least three times. BMM, bone marrow macrophage; SM, spleen macrophage; ROBO4 KO, ROBO4-/-.

Previous report showed that PAF increase in skin induced hair loss of neonates. In order to explain the phenotype observed in Figure 1., we checked the level of PAF in skin from mice with or without ROBO4 deletion using LC-MS assay. We found that mice with ROBO4 deletion exhibited a significantly decrease on PAF concentration in skin for both C16 and C18 (Figure 2A, 2B). To investigate the reason of the decrease of PAF concentration in skin with ROBO4 deletion, we tested the expression of LPCAT1/LPCAT2, two main enzymes used for the synthesis of PAF. Because macrophage was found to be significantly increased in skin of neonate's mice with VLDLR deletion 7, therefore, we detected the expression of LPCAT1/LPCAT2 genes in macrophages differentiated from bone marrow or spleen of mice. As the results showed, the mRNA expression of LPCAT1/LPCAT2 was almost the same (Figure 2C, 2D); however, the expression level of LPCAT1/ LPCAT2 protein significantly down-regulated in macrophage with ROBO4 deletion (Figure 2E, 2F). On the other hand, PAF acetylhydrolase (PAFAH) hydrolyzes PAF, leading to the reduction of PAF concentration 6. It was very difficult to acquire the milk of lactated mice. We then investigated the concentration of PAFAH in serum. We surprisingly found that the concentration of PAFAH increased in serum with ROBO4 deletion comparing to wild type (Figure 2G). Usually, PAF binds to its receptor (PAFR) to activate the downstream signals 4, 5. We thus detected the mRNA and protein expression of PAFR in macrophage with qPCR and Western-blotting. As the results showed that the expression of both mRNA and protein diminished in macrophage with ROBO4 deletion (Figure 2H, 2I). PAF-PAFR signal pathway also involved in the neovascularization, we thus tested the expression of PAFR in retina. Through transcriptome sequencing and Western-blotting analysis, we also observed a reduced expression of PAFR in retina with ROBO4 deletion (Figure 2J, 2K). Interestingly, PAFR expression was significantly enhanced in macrophage with VLDLR deletion compared with that in wild type [Figure 2H]. Moreover, we found that PAFR also down-regulated in ocular tissue for both mRNA and protein (Supplemental Figure 1).Taken together, we found that ROBO4 is associated with the PAF-related pathways.

### ROBO4 deletion partially impaired the mRNA translational efficiency of LPCAT1/LAPCAT2

**Figure 3:**
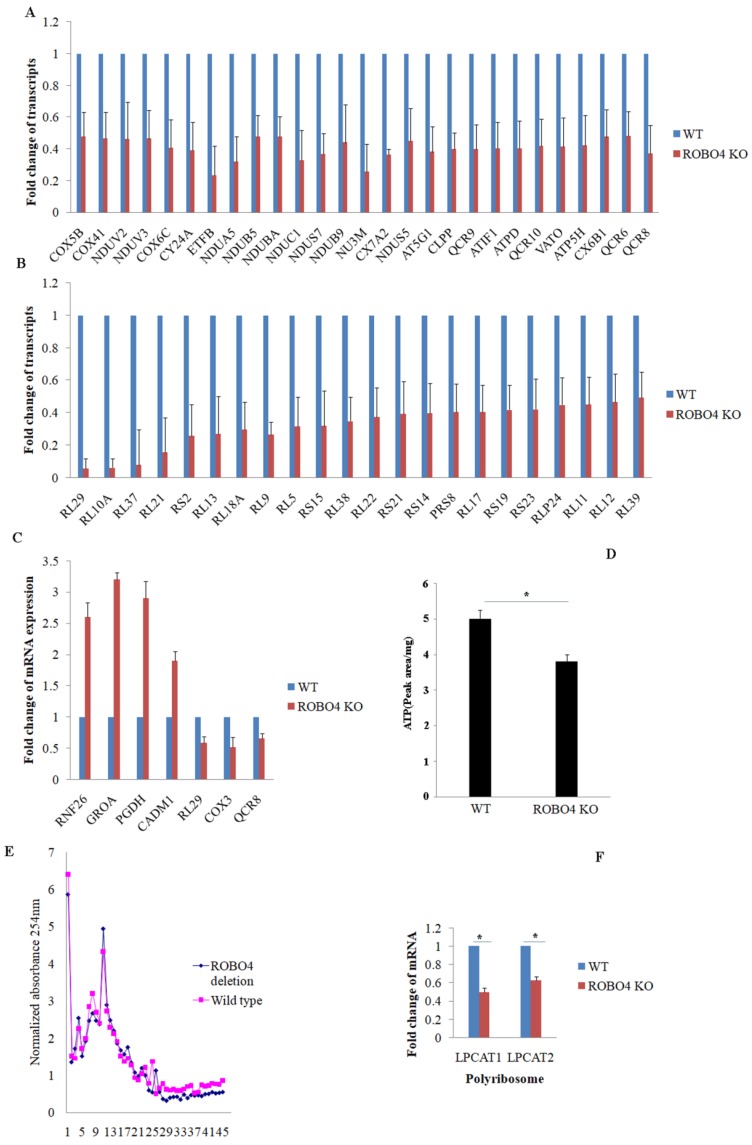
** ROBO4 deletion down-regulated the mRNA expression associated with genes targeted for protein synthesis and oxidative phosphorylation, and reduced ATP synthesis and mRNA translation efficiency of LPCAT1/LPCAT2. A,B**, Part of differential mRNA expression genes in oxidative phosphorylation(A) and in ribosome(B),The expression of specific gene in wild type was set as a 1.**C,** Several differential mRNAs were selected to confirm their expressions via qPCR, beta-actin as internal control. **D**,the concentration of ATP significantly decreased in ROBO4 deletion macrophages; The supernatants from centrifuged macrophages' lysates were acquired and used for the HPLC analysis. The peak area was used to quantify the amount of ATP in cells after comparing to the standard ATP area. **E,F,** Polyribosome(polysome) assay showed the polyribosome slightly decreased in ROBO4 deletion macrophage and the translation efficiency of LPCAT1 and LPCAT2 was significantly reduced in ROBO4 deletion macrophage. E, Macrophage differentiated from both spleen and bone marrow were used in this assay. The OD value in each fraction isolated from the sucrose gradient after centrifuging detected via 254nm. The OD values were normalized to the no protein sample control. F, The amount of mRNA of LPCAT1 and LPCAT2 in polysome was detected by qPCR and GAPDH as an internal reference. Statistical analyses were performed with Student's t-Test and are shown as mean ± standard deviation;*, p<0.05; Experiments repeated at least three times. ROBO4 KO, ROBO4-/-.

To explain the potential mechanism involving in the expression of LPCAT1/LPCAT2, we systematically study the effect of ROBO4 on global RNA expression in macrophage via transcriptome sequencing analysis. From the results of sequencing, we found that ROBO4 deletion changed the expression of various genes (Supplemental data 1). Most genes belonged to the systems of oxidative phosphorylation and ribosome were found to be down-regulated and listed in Figure 3A (oxidative phosphorylation), and Figure 3B (ribosome). In order to confirm the RNA sequence result, we randomly selected genes with different expression levels such as RNF26, GROA, PGDH, CADM1, RL29, COX3 and QCR8 via qPCR.The results of qPCR (Figure 3C) showed a basically consistent trend with the results of RNA sequence. Oxidative phosphorylation and ribosome are responsible for ATP production and protein synthesis, respectively. And enough energy will benefit the protein synthesis. So next, we detected the level of ATP in macrophage with HPLC. We found that the ATP concentration significantly diminished in ROBO4 deletion macrophage compared to that of wild type (Figure 3D). Polyribosome assay is usually used to assess the translational efficiency of specific gene. Here, we isolated the polyribosome in macrophage lysates using the sucrose gradient analysis and extracted the total RNAs from the polyribosome fraction. We then detected the level of mRNAs of LPCAT1/LPCAT2 using qPCR, GAPDH as an internal reference. As expected, ROBO4 deletion slightly decreased the level of total polysome (Figure 3E), which well matches the results of RNA sequence. We also observed the level of LPCAT1/LPCAT2 mRNA binding to the polysome significantly diminished in ROBO4 deletion macrophage (Figure 3F). The translational efficiency of specific gene is positively correlated with the binding ability to the polysome. We therefore argued that the decreased efficiency of LPCAT1/LPCAT2 mRNA translation reduced level of their protein in ROBO4 deletion macrophage. However, how ATP change affects PAF production need further confirm.

### Mice with ROBO4 deletion decreased LPS-mediated inflammation

**Figure 4:**
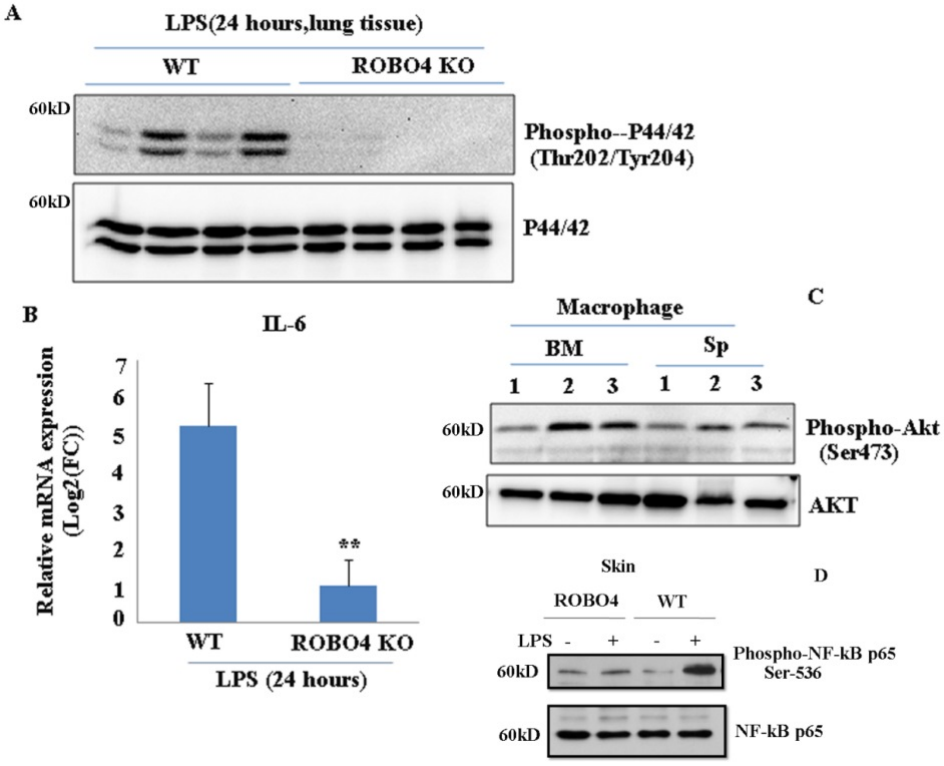
ROBO4 deletion suppressed LPS-induced IL-6 expression as well as the phosphorylation of p44/42, NF-kB p65 , but enhanced the phosphorylation of AKT in lung tissue. A, ROBO4 deletion suppressed LPS induced the phosphorylation of p44/42 at the Thr202 and Tyr204 in lung tissues; B, ROBO4 deletion suppressed LPS induced IL-6 expression in eye tissues. 200ug LPS was I.P injected into four wild type C57BLs or four ROBO4 deletions (ROBO4 KO) C57BLs for 24 hours. Then eye ocular and lung tissues were dissected, and used for total RNA extraction and protein extraction. C, Phosphorylation of AKT at Ser473 enhanced in ROBO4 deletion macrophage, but diminished in VLDLR macrophage. D,Wild type C57BLs and ROBO4 deletion(KO) (three mice for each type) was I.P infected with LPS for 24 hours, skin tissue was used for the phosphorylation assay with anti-phospho-NF-kB p65(Ser-536) antibody and NF-kB p65 antibody was used as a loading control. Macrophage isolated from bone marrow or spleen was differentiated by M-CSF. After 12 days culture, cells were harvested and used for the Western-blotting assay with designated antibodies. 1, VLDLR deletion; 2, ROBO4 deletion; 3, wild type.

To investigate whether ROBO4 deletion also affects other inflammation associated signal pathways, we stimulated the ROBO4 deletion mice and wild type mice with LPS for 24 hours via intraperitoneal injection. LPS is an important inducer of inflammation. It activates various signaling pathways such as NF-kB, and Erk ,in turn promotes a variety of genes expression including interleukin 6 (IL-6) .Hence, we then tested the IL-6 expression and the phosphorylation of AKT ,p65 and p44/42. We found the expression of IL-6 significantly reduced in lung tissues with ROBO4 deletion compared to that of wild-type (Figure 4B). The level of p44/42 and p65 phosphorylation also decreased in ROBO4 deletion mice (Figure 4A, 4D). However, the level of AKT phosphorylation enhanced in ROBO4 deletion mice in response to LPS stimulation (Figure 4C).These results supported that ROBO4 is also a key regulator of LPS induced inflammation. Then, we further treated the M-CSF-defferentiated macrophage isolated from the spleen with 1μM U0126, an inhibitor of P44/42 kinase, for 24 hours. After that, cells were used for western blotting or PAF extraction. We found that inhibition of P44/42 could significantly decreased the level of PAF-C16 but not the PAF-C18 (Supplemental Figure 3 B). The efficiency of inhibitor was confirmed by checking the level of phosphorylation of P44/42 (Supplemental Figure 3A).

## Discussion

Roundabout (Robo) receptors play key roles in development of the nervous system 13. As an endothelial-specific Robo receptor, ROBO4 maintains the vascular integrity by inhibiting VEGF receptor activating pathways 9, 10, and also cooperates with Cxcr4 to regulate the localization of hematopoietic stem cell 11. Recently, ROBO4 was proved to be involved in the inflammation inhibition 12. Hence, ROBO4 might play diverse functions during the progress of physiological and pathological events. Here we reported ROBO4 modified PAF-associated signals. PAF is a potent pro-inflammatory phospholipid and thus plays roles in many diseases, including anaphylaxis, sepsis, acute respiratory distress syndrome, bronchial asthma, and G-protein-coupled PAF receptor (PAFR) mediated inflammatory signals 1, 5, 7. PAF also promotes neovascularization 4 and tumorgenesis 8. The deletion of VLDLR in mice not only causes hair loss phenotype on the skin of lactating pups 6, but also leads to severe neovascularization in retina 14, 15. On the other hand, ROBO4 can stabilize blood vessel. Therefore, based on our currently results, we argued that PAF balance disrupted by VLDLR deletion can be offset by ROBO4 deletion.

Another interestingly finding was that most of the genes involving in oxidative phosphorylation (OXPHOS), ribosome and ubiquinone biosynthesis were all significantly down-regulated in ROBO4 deletion macrophage (Figure 3A, B; Supplemental Table 1). OXPHOS, a very complex but important system, provides adequate cellular energy currency adenosine 5′-triphosphate (ATP) to support many cellular events 16. This result was consistent with the change of ATP concentration (Figure 3D). Protein synthesis needs large amount of energies. Thus, impaired energy synthesis might reduce the translational efficiency of mRNAs. Moreover, the genes response for the ribosome assembly also significantly down regulated (Figure 3A, B; Supplemental Table 1). Ribosome is a complex structure for mRNA translation 17. Highly translated mRNAs usually were bound by many Ribosomes also named polyribosome 17, 18. That's to say, two key factors for protein synthesis are impaired in ROBO4 deletion macrophage. LPCAT1/LPCAT2 expression might be an example for this deduction. There are also reports showed slowing down mitochondrial metabolism and protein synthesis benefit the lifespan in several species 19. Therefore, ROBO4 might also link to the regulation of species' lifespan.

Interleukin-6 (IL-6) is a multifaceted inflammatory cytokine produced by various kinds of cell types. Its plays critical roles in immune responses, hematopoiesis and angiogensis 20. Our results and other previous reported data showed ROBO4 is responsible for LPS-stimulated IL-6 production (Figure 4B) 20. In response to LPS stimulation, the decrease of phosphorylation of P42/44 at Thr202 and Tyr204 (Figure 4A), NF-kB p65 at Ser536 and the enhancement of phosphorylation of AKT protein (Figure 4C) in ROBO4 deletion mice indicates that those pathways might participate in the ROBO4-mediated inflammation. Previous report showed that LPS stimulation can induce the expression of LPCAT2 21. Hence, it is possible that ROBO4 deletion might prevent LPCAT2 expression in response to LPS stimulation, in turn, regulating PAF-associated inflammation.

## Conclusion

Taken together, ROBO4 deletion regulates PAF-associated inflammation partially through interfering with the mRNA translation of LAPCAT1/ LPCAT2 and the expression of PAFR.

## Supplementary Material

Supplementary figures.

Supplementary table.

## Figures and Tables

**Table 1:** Primers and Usages

Gene Name	Sequence(5'-3')	Usage
RNF26	GTACTTACCAGTCTTCTGCACTTG	qPCR confirmation
	TGGCCATTGGAGACCATGTTCAG	
PGDH	AGGTAGCATTGGTGGATTGGAATC	qPCR confirmation
	TCATTGTTCACGCCTGCATTGTTG	
CADM1	AGCAGTGAACTCAAAGTGTCACTG	qPCR confirmation
	TGCTGGCCATGGCAGTACAGTTG	
RL29	AAGAAGATGCAGGCCAACAATG	qPCR confirmation
	GTTTGGACCTTAGGCTTCGGTTG	
COX3	AGCCCTCCTTCTAACATCAGGTC	qPCR confirmation
	AGTGTGGTGGCCTTGGTAGGTTC	
QCR8	GGATACGGCACGTGATCTCCTAC	qPCR confirmation
	TGTAGATCAGGTAGACCACTAC	
LPCAT1	TGCTCAAGGCCATCATGCGCACC	Polyribosome assay
	TCTCTGCTCTCTGCCTTCATCAC	
LPCAT2	TAGTAGCTGGGCTGCCTTCGCTGG	Polyribosome assay
	AACAGGAGCGATTAGTACAAGTAC	
GAPDH	AGCAGTCCCGTACACTGGCAAAC	qPCR confirmation
	TCTGTGGTGATGTAAATGTCCTCT	
