# Supplementary material for: ROBO4 deletion ameliorates PAF-mediated skin inflammation via regulating the mRNA translation efficiency of LPCAT1/LPCAT2 and the expression of PAF receptor
Source: Int J Biol Sci. 2020 Feb 4;16(6):1086–95. doi: 10.7150/ijbs.35797 (PMC7053341; doi:10.7150/ijbs.35797)
Supplement: Supplementary file 1 — Supplementary figures. [file ijbsv16p1086s1.pdf]

A

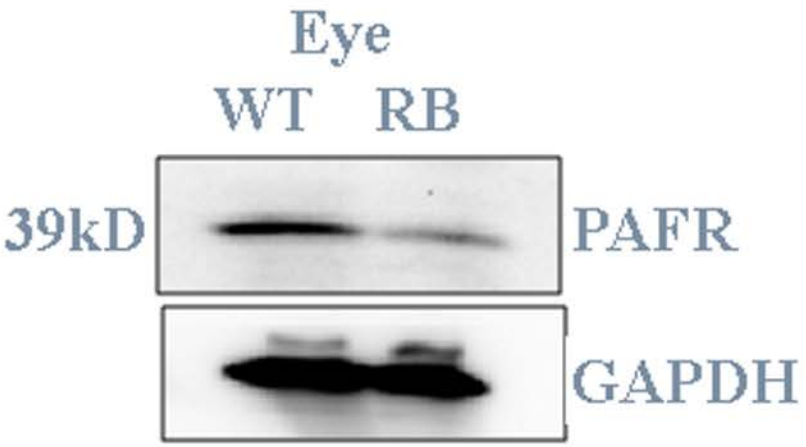

B

Genes	logFC	logCPM	PValue	FDR
ENSMUSG00000056529 PAF Receptor	-3.58064	1.604834	3.15E-11	5.58E-08

A

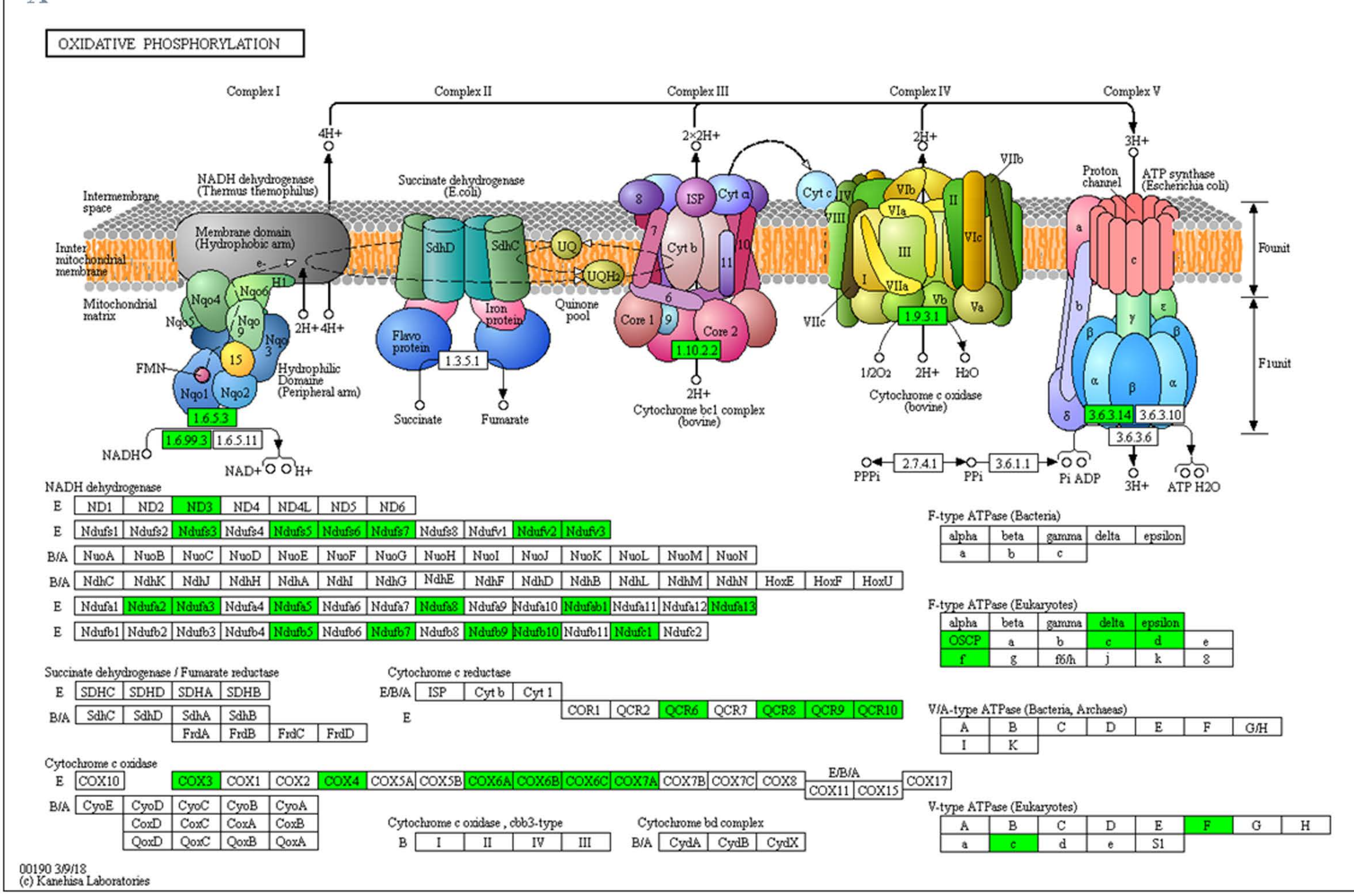
OXIDATIVE PHOSPHORYLATION
Complex I
Complex II
Complex III
Complex IV
Complex V
4H+
2×2H+
2H+
3H+
NADH dehydrogenase (Thermus themophilus)
Succinate dehydrogenase (E.coli)
Intermembrane space
Innter mitochondrial membrane
Mitochondrial matrix
Membrane domain (Hydrophobic arm)
Hydrophilic Domaine (Peripheral arm)
FMN
Nqo4
Nqo5
Nqo6
Nqo1
Nqo2
Nqo3
Nqo9
15
H1
e-
2H+ 4H+
1.6.5.3
1.6.99.3
1.6.5.11
NADH
NAD+
H+
SdhD
SdhC
UQ
UQH2
Quinone pool
Flavo protein
Iron protein
1.3.5.1
Succinate
Fumarate
ISP
Cyt c1
Cyt b
Core 1
Core 2
1.10.2.2
2H+
Cytochrome bc1 complex (bovine)
Cyt c
VIIb
VIb
VIa
III
II
VIc
VIIa
VIIc
VIII
IV
Vb
Va
1.9.3.1
1/2O2
2H+
H2O
Cytochrome c oxidase (bovine)
Proton channel
ATP synthase (Escherichia coli)
Fo unit
F1 unit
3.6.3.14
3.6.3.10
3.6.3.6
PPPi
2.7.4.1
PPi
3.6.1.1
Pi ADP
3H+
ATP H2O
NADH dehydrogenase
E ND1 ND2 ND3 ND4 ND4L ND5 ND6
E Ndufs1 Ndufs2 Ndufs3 Ndufs4 Ndufs5 Ndufs6 Ndufs7 Ndufs8 Ndufv1 Ndufv2 Ndufv3
B/A NuoA NuoB NuoC NuoD NuoE NuoF NuoG NuoH NuoI NuoJ NuoK NuoL NuoM NuoN
B/A NdhC NdhK NdhJ NdhH NdhA NdhI NdhG NdhE NdhF NdhD NdhB NdhL NdhM NdhN HoxE HoxF HoxU
E Ndufa1 Ndufa2 Ndufa3 Ndufa4 Ndufa5 Ndufa6 Ndufa7 Ndufa8 Ndufa9 Ndufa10 Ndufab1 Ndufa11 Ndufa12 Ndufa13
E Ndufb1 Ndufb2 Ndufb3 Ndufb4 Ndufb5 Ndufb6 Ndufb7 Ndufb8 Ndufb9 Ndufb10 Ndufb11 Ndufc1 Ndufc2
Succinate dehydrogenase / Fumarate reductase
E SDHC SDHD SDHA SDHB
B/A SdhC SdhD SdhA SdhB
FrdA FrdB FrdC FrdD
Cytochrome c reductase
E/B/A ISP Cyt b Cyt 1
E COR1 QCR2 QCR6 QCR7 QCR8 QCR9 QCR10
Cytochrome c oxidase
E COX10 COX3 COX1 COX2 COX4 COX5A COX5B COX6A COX6B COX6C COX7A COX7B COX7C COX8
E/B/A COX11 COX15 COX17
B/A CyoE CyoD CyoC CyoB CyoA
CoxD CoxC CoxA CoxB
QoxD QoxC QoxB QoxA
Cytochrome c oxidase , cbb3-type
B I II IV III
Cytochrome bd complex
B/A CydA CydB CydX
F-type ATPase (Bacteria)
alpha beta gamma delta epsilon
a b c
F-type ATPase (Eukaryotes)
alpha beta gamma delta epsilon
OSCP a b c d e
f g f6/h j k 8
V/A-type ATPase (Bacteria, Archaeas)
A B C D E F G/H
I K
V-type ATPase (Eukaryotes)
A B C D E F G H
a c d e S1
00190 3/9/18
(c) Kanehisa Laboratories

B

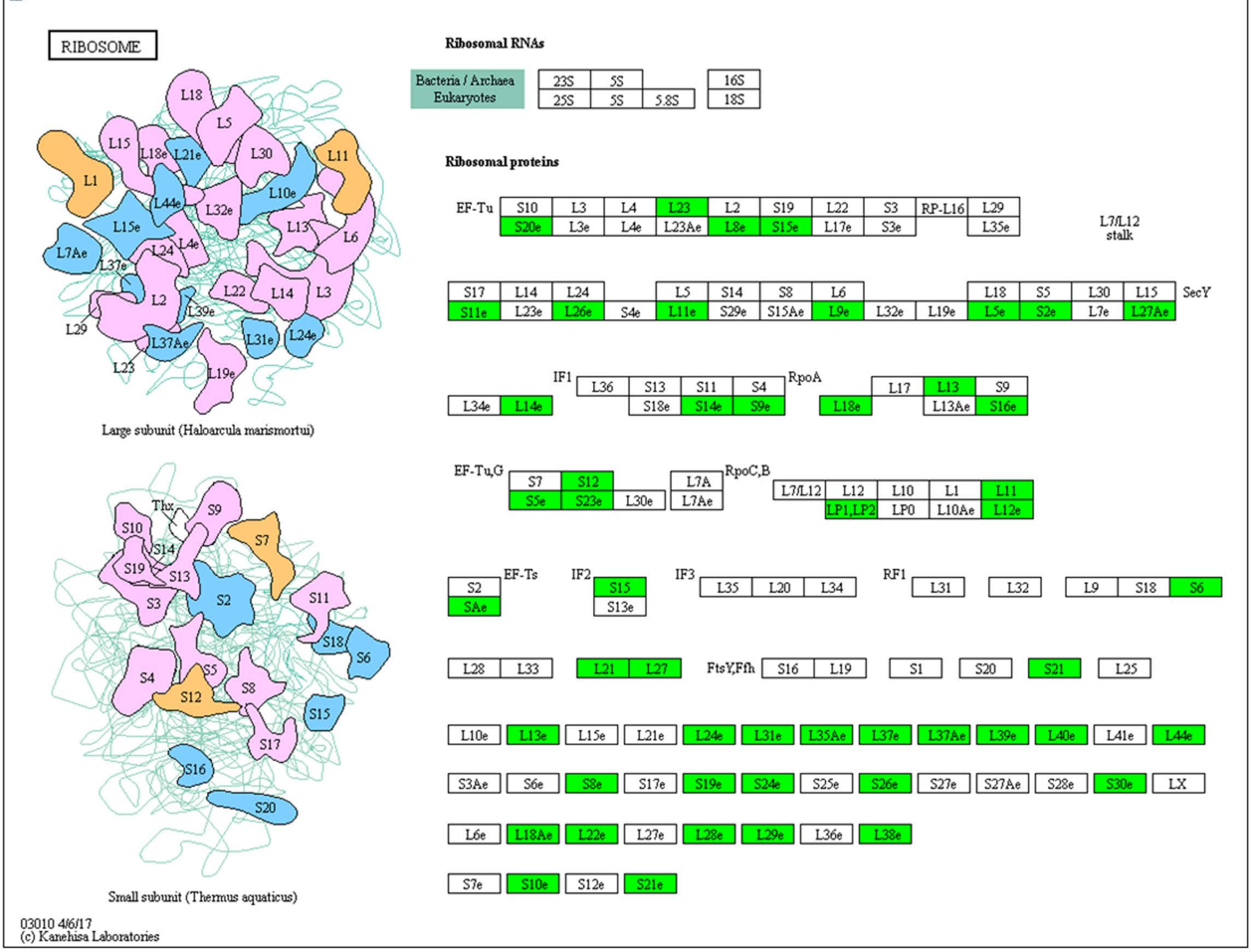
RIBOSOME
Large subunit (Haloarcula marismortui)
Small subunit (Thermus aquaticus)
Ribosomal RNAs
Bacteria / Archaea
Eukaryotes
23S 5S 16S
25S 5S 5.8S 18S
Ribosomal proteins
EF-Tu
S10 L3 L4 L23 L2 S19 L22 S3 RP-L16 L29
S20e L3e L4e L23Ae L8e S15e L17e S3e L35e
L7/L12 stalk
S17 L14 L24 L5 S14 S8 L6 L18 S5 L30 L15 SecY
S11e L23e L26e S4e L11e S29e S15Ae L9e L32e L19e L5e S2e L7e L27Ae
IF1
L36 S13 S11 S4
RpoA
L17 L13 S9
L34e L14e S18e S14e S9e L18e L13Ae S16e
EF-Tu,G
S7 S12 L7A
S5e S23e L30e L7Ae
RpoC,B
L7/L12 L12 L10 L1 L11
LP1,LP2 LP0 L10Ae L12e
S2 EF-Ts IF2 S15 IF3 L35 L20 L34 RF1 L31 L32 L9 S18 S6
SAe S13e
L28 L33 L21 L27 FtsY,Ffh S16 L19 S1 S20 S21 L25
L10e L13e L15e L21e L24e L31e L35Ae L37e L37Ae L39e L40e L41e L44e
S3Ae S6e S8e S17e S19e S24e S25e S26e S27e S27Ae S28e S30e LX
L6e L18Ae L22e L27e L28e L29e L36e L38e
S7e S10e S12e S21e
03010 4/6/17
(c) Kanehisa Laboratories

A

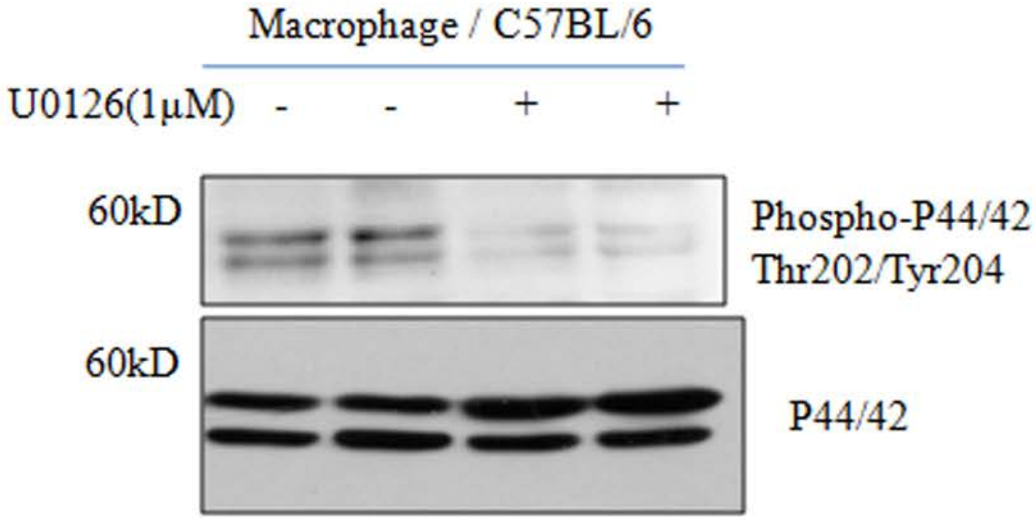
Macrophage / C57BL/6
U0126(1μM) - - + +
60kD
Phospho-P44/42
Thr202/Tyr204
60kD
P44/42

B

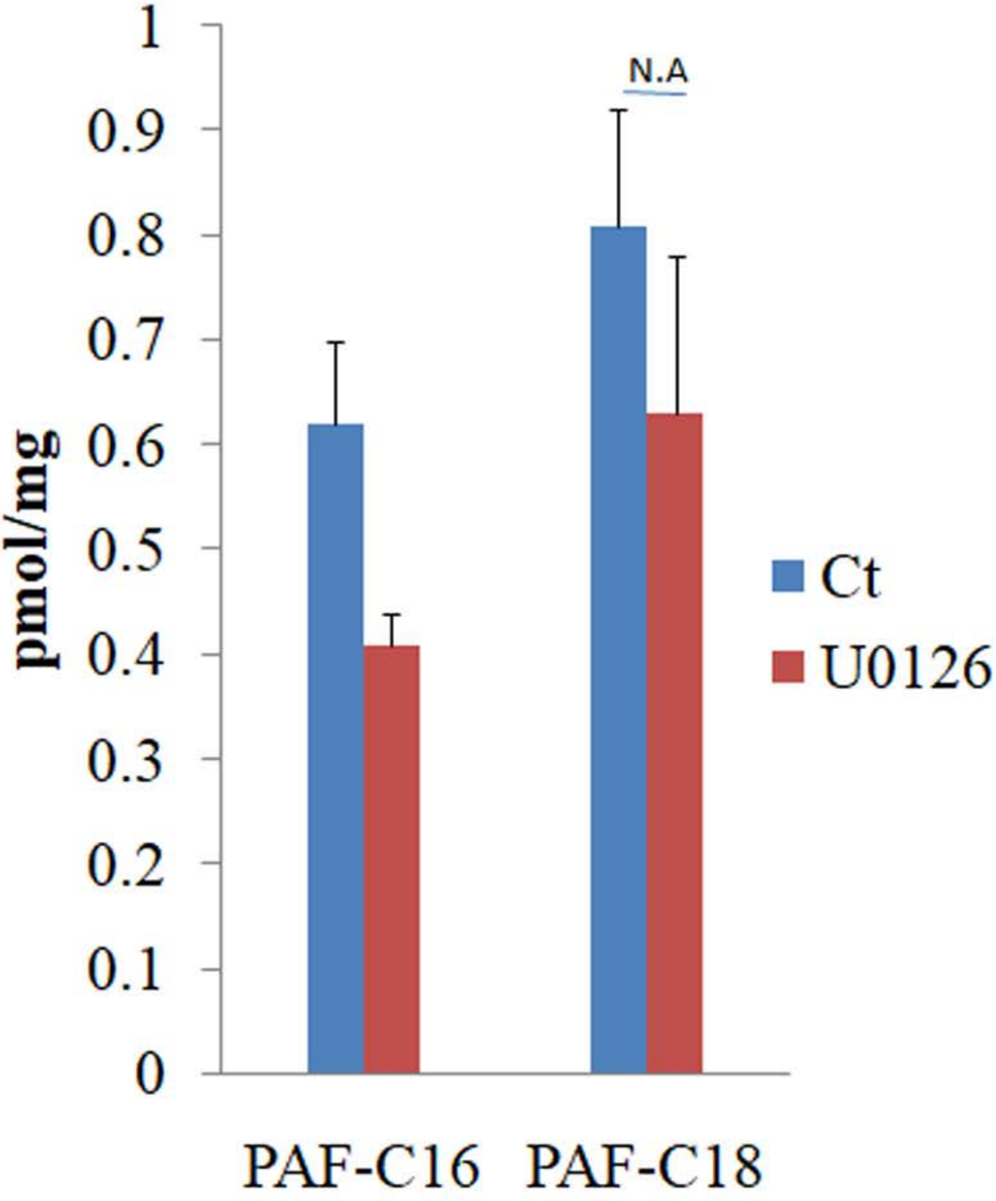
1
0.9
0.8
0.7
0.6
0.5
0.4
0.3
0.2
0.1
0
pmol/mg
N.A
Ct
U0126
PAF-C16
PAF-C18
